# Habitual fish intake negatively correlates with prevalence of frailty among patients with rheumatoid arthritis

**DOI:** 10.1038/s41598-021-84479-0

**Published:** 2021-03-03

**Authors:** Hiroto Minamino, Masao Katsushima, Mie Torii, Motomu Hashimoto, Yoshihito Fujita, Kaori Ikeda, Wataru Yamamoto, Ryu Watanabe, Kosaku Murakami, Koichi Murata, Kohei Nishitani, Masao Tanaka, Hiromu Ito, Koichiro Ohmura, Hidenori Arai, Nobuya Inagaki, Shuichi Matsuda

**Affiliations:** 1 Department of Diabetes, Endocrinology and Nutrition, Graduate School of Medicine, Kyoto University, Kyoto, Japan; 2 Japan Society for the Promotion of Science, Tokyo, Japan; 3 Department of Rheumatology and Clinical Immunology, Graduate School of Medicine, Kyoto University, Kyoto, Japan; 4 Department of Human Health Sciences, Graduate School of Medicine, Kyoto University, Kyoto, Japan; 5 Department of Advanced Medicine for Rheumatic Diseases, Graduate School of Medicine, Kyoto University, Kyoto, Japan; 6 Department of Health Information Management, Kurashiki Sweet Hospital, Kurashiki, Japan; 7 Department of Orthopedic Surgery, Graduate School of Medicine, Kyoto University, Kyoto, Japan; 8 National Center for Geriatrics and Gerontology, Obu, Japan

**Keywords:** Endocrinology, Health care, Rheumatology, Risk factors

## Abstract

Frailty is a geriatric syndrome characterized by anabolic-catabolic imbalance and multisystem dysregulation resulting in increased adverse health outcomes, and is closely related with dietary habits in the general population. Although chronic inflammatory diseases are thought to accelerate development of frailty, correlations between rheumatoid arthritis (RA), frailty and dietary habits have not been examined. We performed a cross-sectional study using our cohort database (KURAMA cohort), and classified 306 participants into three groups (robust, prefrail and frail) according to the Study of Osteoporotic Fracture (SOF) criteria. Multivariate logistic analysis revealed that the presence of frailty/prefrailty was significantly correlated with the disease activity score (DAS28-ESR) (OR 1.70 (1.30–2.22), p < 0.0001). Additional analyses of frailty and food intake showed that 5 foods (fish, meat, milk, vegetables and fruits) of 20 groups on the questionnaire were inversely associated with the prevalence of frail/prefrail categories. In multivariate analysis with the five nutrients, fish intake (> two times a week) was an independent covariate negatively correlated with frailty/prefrailty (OR 0.35 (0.19–0.63), p = 0.00060). In conclusion, habitual fish intake may play a key role in nutritional intervention to prevent progression of frailty and RA.

## Introduction

Rheumatoid arthritis (RA) is a chronic autoimmune disease characterized by joint destruction; cytokines such as tumor necrosis factor-α (TNF-α) and interleukin-6 (IL-6) play key roles in systemic inflammation^1^. RA patients show increased prevalence of comorbidities (i.e., cardiovascular disease, sarcopenia, osteoporosis and bone fracture)^2,3^, and declining functional status and body weight loss are reported as risk factors for mortality^4,5^. Especially, the elderly with RA often have multimorbidity and drug intolerance, and tend to experience functional decline due to poor treatment response as well as limited therapeutic options^6,7^.

Frailty, a key concept in geriatric medicine, is a state of compromised physiologic reserve and increased risk for adverse outcomes and mortality^8^. Frailty has a complex multifactorial etiology, and chronic inflammation is a crucial factor that increases catabolism and interferes with homeostatic signaling^9,10^. Serum levels of inflammatory molecules are elevated in the frail elderly^11^, and it has been proposed that several inflammatory diseases accelerate anabolic resistance and the development of frailty^12^. Although RA is a representative of chronic inflammatory disease, little is known of the interaction between frailty and RA-related factors such as current disease activity and therapeutic agents.

Recently, a number of studies have reported the impact of dietary habits on frailty and several other diseases. Nutritional interventions such as protein supplementation were found to improve physical function of the frail elderly in several clinical trials^13^. In the RA population, seafood, fruits and vegetables have beneficial effects on disease activity^14^, whereas high-fat diets have the opposite effect by leading to a pro-inflammatory response^15^. Even though several nutrients are expected to have therapeutic effects both on frailty and inflammatory diseases, clinical correlations between dietary habits and frailty with RA have not been examined.

In the current study, we conducted a cross-sectional study using the Kyoto University RA Management Alliance cohort (KURAMA) to clarify the relationships among frailty, dietary habits and disease activity of RA patients.

## Material and methods

### Study population and settings

In the present study, we conducted a cross-sectional study of female outpatients with RA from the KURAMA (Kyoto University Rheumatoid Arthritis Management Alliance) cohort database^16^. A total of 388 outpatients with RA who were over 18 years of age and visited the Kyoto University Hospital between 1st May and 31st December 2014 were recruited for the study. After we excluded 82 patients for lack of a complete data set or incomplete answers to the food intake questionnaire, we included the remaining 306 patients in the study. This research complied with the Declaration of Helsinki and was approved by the Medical Ethics Committee of Kyoto University Graduate School and Faculty of Medicine (Approval number: R0357). Informed consent was obtained from all subjects, including the use of human blood samples and data.

### Evaluation of frailty and frailty-related parameters

We assessed frailty status of the subjects using the Study of Osteoporotic Fractures (SOF) criteria as previously reported^17^. The SOF index comprises the following three components: 1. weight loss (5% or more reduction in the previous year), 2. chair stands (inability to rise from a chair 5 times without using hands) and 3. reduced energy levels detected by the question: “Do you feel full of energy?” from the Geriatric Depression Scale. Patients with the presence of 0, 1, and 2 or 3 components were defined as Robust, Prefrail, and Frail, respectively. To assess frailty-related physical activity parameters, we evaluated hand grip strength, walking speed and skeletal muscle mass. We measured hand grip strength using a JAMAR digital hand dynamometer (Patterson Medical, Bolingbrook, IL), and evaluated 6-m walking speed by a portable gait rhythmogram (MG-M1110: LSI Medience Co., Tokyo, Japan). Skeletal muscle mass was assessed by bioelectrical impedance Analysis (Inbody 720: Biospace Co., Ltd., Seoul, Korea). Skeletal muscle index (SMI) was calculated as the skeletal muscle mass in kilograms divided by height in meters squared. We used self-reported questionnaire forms for a history of falls and bone fractures in the previous year.

### Assessment of dietary habits

Dietary habits of RA patients were obtained from the self-reported questionnaire as described previously^14,18^. This questionnaire consisted of 20 items about the intake frequency of 1. staple food (rice, bread or noodles) for breakfast, 2. staple food for lunch, 3. staple food for dinner, 4. meat, 5. fish, 6. *tofu* (soybean curd), 7. eggs, 8. milk, 9. vegetables, 10. fruits, 11. deep-fried foods, 12. cakes, 13. juice or isotonic drinks, 14. junk foods, 15. sweets like candies and chocolates, 16. frozen foods, 17. pickles, 18. ham, sausage or *kamaboko* (boiled fish paste), 19. *miso* soup (fermented soybean paste) and by the participants. alcohol. The intake frequency was collected for each item on the basis of the following 8 categories: 1 =  < 1 time/month, 2 = 1–3 times/month, 3 = 1–2 times/week, 4 = 3–4 times/week, 5 = 5–6 times/week, 6 = 1 time/day, 7 = 2 times/day, 8 = 3 times/day.

### Estimation of RA-related factors

The disease activity and physical disability of RA were evaluated by the 28-Joint RA Disease Activity Score (DAS28-ESR), Steinbrocker’s stage and the health assessment questionnaire disability index (HAQ). Baseline laboratory data were assessed including hemoglobin (Hb), albumin (Alb), and C-reactive protein (CRP). The data on Current RA medications including Methotrexate (MTX), prednisolone (PSL), Tumor necrosis factor-α (TNF-α) inhibitors, Interleukin-6 (IL-6) receptor inhibitors and cytotoxic T-lymphocyte antigen 4 (CTLA4) immunoglobulin were collected from the KURAMA database.

### Statistical analysis

Data on continuous variables, dietary habits and categorical variables are expressed using median (interquartile), median (range) and numbers (%), respectively. To compare characteristics of RA patients divided into three groups by frailty status, we carried out a Steel–Dwass test for continuous variables or a Fisher’s exact test for categorical variables. To analyze the relationship between the frequency of each food intake and the prevalence of prefrail or frail, we used the Cochran-Armitage trend test.

For identification of RA-related contributing factors to frailty, we first adopted univariate logistic regression analysis. We constructed a dummy variable as follows: 0 = robust and 1 = prefrailty or frailty, and following analyses were carried out with its frailty variable as a dependent variable. After selecting variables detected as significant variables in the univariate analysis that were considered to be clinically relevant, we conducted multivariate logistic regression analysis. To determine the dietary factors that contribute to frailty status, further analysis was carried out. A dummy variable was constructed with 0 and 1: fish and meat dish, 0 = low frequency (≤ 2 times/weeks) and 1 = high frequency (3 times/week ≤); milk, vegetable and fruits, 0 = low frequency (≤ 6 times/week) and 1 = high frequency (1 time/day ≤). As the distribution of intake frequency of each food differed greatly, we redistributed intake frequency into two categories based on the medians. We then adopted multivariate logistic regression analysis using these variables as well as RA-related contributing factors. Furthermore, the same analysis was conducted in elderly RA patients aged 65 years and older (n = 147) because clinical definitions of frailty are generally used for the elderly who are over 65 years of age. In addition, we also constructed another logistic regression model, a forward stepwise logistic regression model, to confirm the independent variables (P for enter < 0.1, and for exit > 0.1). Statistical significance was analyzed using JMP14 (SAS Institute Inc., Cary, NC, USA) and P values < 0.05 were considered significant.

## Results

### Baseline characteristics of the study population

A total of 306 female patients with RA were enrolled in the following analyses. Characteristics of the patients are described in Table 1. The mean age and disease duration of RA were 61.5 years and 13.7 years, respectively. Using the SOF index, 23.2% were identified as frail, 32.7% as prefrail, and 44.1% as robust; the prevalence of prefrailty or frailty was higher than that in general population^19,20^*.* Regarding the current RA medications being used by the participants in this study, 72.5%, 28.6%, and 43.8% of patients were treated by MTX, PSL, and biological agents, respectively.

**Table 1:** Characteristics of study population.

Characteristics	RA patients
	(*N* = 306)
Age, years	63.5 (53–71)
**Body composition and physical activity parameters**	
Body mass index, kg/m^2^	21.2 (19.3–23.7)
Skeletal mass index, kg/m^2^	5.7 (5.2–6.2)
Right hand grip, kg	15.5 (9.5–21.2)
Light hand grip, kg	15.0 (9.0–20.7)
Walking speed, m/s	1.0 (0.8–1.2)
**Frailty score (SOF index)**	
0, *n* (%)	135 (44.1)
1, *n* (%)	100 (32.7)
2, *n* (%)	71 (23.2)
Any fall in previous year, *n* (%)	60 (19.6)
Any fracture in previous year, *n* (%)	18 (5.9)
**RA disease characteristics**	
Duration, years	9.0 (3–21)
DAS28-ESR	2.8 (2.1–3.5)
HAQ score	0.5 (0–1)
Stage*	3 (2–4)
Class*	2 (1–2)
**Current RA medications**	
Methotrexate use, *n* (%)	222 (72.5)
Prednisolone use, *n* (%)	88 (28.6)
Biological agent use, *n* (%)	134 (43.8)
**Laboratory data**	
Hemoglobin, g/dL	12.6 (11.8–13.4)
Albumin, g/dL	3.9 (3.7–4.1)
CRP, mg/dL	0.1 (0–0.3)
Dietary habits**	
Staple food for dinner	6 [1–8]
Fish dishes	4 [1–7]
Meat dishes	4 [1–8]
Egg dishes	4 [1–8]
Vegetable dishes	6 [1–8]
Fruits	6 [1–8]
Milk	6 [1–8]

Data are expressed as median (interquartile) for continuous variables, median (range) for dietary habits scores, and numbers (%) for categorical variables.

*RA* rheumatoid arthritis, *SOF* index Study of Osteoporotic Fractures index, *DAS28-ESR* 28-joint Disease Activity Score using erythrocyte sedimentation rate, *HAQ* health assessment questionnaire, *TNF* tumor necrosis factor, *IL-6* interleukin-6, *CTLA4* cytotoxic T-lymphocyte antigen 4, *CRP* C-reactive protein.

*Steinbrocker's Stage and Class.

**Dietary habit scores: 0 = seldom, 1 =  < 1 time/month, 2 = 1–3 times/month, 3 = 1–2 times/week, 4 = 3–4 times/week, 5 = 5–6 times/week, 6 = 1 time/day, 7 = 2 times/day, 8 = 3 times/day.

### Comparison of characteristics among robust, prefrail and frail subjects

To characterize differences among the frailty stages, three groups were compared: robust, prefrail, and frail (Table 2). The average age and duration of RA in the frail group were significantly higher than those in the robust group. As the frailty stage increased, the factors associated with frailty including skeletal muscle mass, strength of hand grip and walking speed decreased; by contrast, the levels of RA disease activity including DAS28-ESR increased. The number of bone fractures, which is one of the more serious outcomes of frailty, was significantly increased in the frail group. Regarding the current therapeutic drugs, the user rate of MTX was higher in the robust group than that in the frail group. On the contrary, the user rate of PSL was lower in the robust group than in the frail group.

**Table 2:** Characteristics of RA patients by Frailty status.

Frailty score (SOF index)	Robust	Prefrail	Frail	*P* value
	0	1	2 or 3	
(N = 306)	*n* = 135 (44.1%)	*n* = 100 (32.7%)	*n* = 71 (23.2%)	
Age, year	62.0 (49.0–69.0)	64.0 (57.0–71.0)	67.0 (59.0–74.0)	0.0012
Body mass index, kg/m^2^	21.2 (19.5–23.8)	21.7 (19.2–24.8)	20.7 (18.8–22.9)	0.26
**Factors associated with frailty**				
Skeletal mass index, kg/m^2^	5.87 (5.35–6.32)	5.69 (5.16–6.27)	5.35 (4.88–5.94)	0.0054
Right hand grip, kg	18.1 (12.9–24.2)	15.7 (9.7–20.6)	10.0 (6.4–14.7)	< 0.0001
Light hand grip, kg	17.8 (11.9–22.9)	15.1 (9.6–20.6)	9.0 (5.5–14.8)	< 0.0001
Walking speed, m/s	1.12 (0.98–1.25)	1.05 (0.81–1.17)	0.73 (0.62–0.96)	< 0.0001
Any fall in previous year, *n* (%)	23 (17.0)	16 (16.0)	21 (29.6)	0.058
Any fracture in previous year, *n* (%)	0 (0)	9 (9)	9 (12.7)	0.0181
**Laboratory data**				
Hemoglobin, g/dL	12.9 (12.0–13.5)	12.6 (11.8–13.2)	12.1 (11.2–13.0)	0.0023
Albumin, g/dL	4.0 (3.8–4.2)	3.9 (3.7–4.1)	3.9 (3.6–4.1)	0.0139
CRP, mg/dL	0.1 (0–0.2)	0.1 (0–0.3)	0.1 (0–0.4)	0.037
RA disease characteristics				d
Disease duration, year	7.0 (3.0–20)	8.0 (3.0–19.8)	15.0 (7–27)	0.0019
DAS28-ESR	2.32 (1.84–3.15)	2.78 (2.08–3.68)	3.38 (2.78–4.23)	< 0.0001
HAQ	0.13 (0–0.50)	0.63 (0.25–1.13)	1.25 (0.75–1.63)	< 0.0001
Stage	1 (1–2)	2 (2–2)	2 (2–2)	0.026
Class	3 (2–4)	3 (2–4)	3 (2–4)	< 0.0001
**Current therapeutic agent**				
MTX use, *n* (%)	109 (80.7)	70 (70.0)	43 (60.6)	0.0016
Biological agent use, *n* (%)	61 (45.2)	47 (47.0)	26 (36.6)	0.31
Prednisolone use, *n* (%)	28 (20.7)	30 (30.0)	30 (42.2)	0.0012

Data are expressed as median (interquartile) for continuous variables and numbers (%) for categorical variables.

*RA* rheumatoid arthritis, *SOF* index Study of Osteoporotic Fractures index, *CRP* C-reactive protein, *DAS28-ESR* 28-joint Disease Activity Score using erythrocyte sedimentation rate, *HAQ* health assessment questionnaire, *MTX* methotrexate.

### Disease activity of RA is independently associated with frailty status

To learn which factors contribute to prefrail and frail status, we carried out logistic analysis. In univariate analysis, age, duration of RA, DAS28-ESR, HAQ, and PSL use were positively associated with the presence of prefrailty or frailty (Table 3 left). In contrast, MTX use, hand grip strength, and walking speed were negatively associated with the condition. We then performed multivariate logistic analyses using the following covariates: age, duration of RA, DAS28-ESR, MTX use, PSL use, and biological agents use. Hand grip strength and walking speed were not included in the analysis because these factors may be constitutive of frailty itself. HAQ was not also included because this factor may be the outcome of frailty rather than the cause of frailty. As a result, DAS-28-ESR (OR 1.70: 95% CI 1.30–2.22) and MTX use (OR 0.47: 95% CI 0.26–0.84) were identified as independent covariates associated with prefrailty or frailty (Table 3 right).

**Table 3:** Logistic analysis for RA patients with prefrailty or frailty.

Variables	Univariate			Multivariate		
	OR	95% CI	*P* value	OR	95% CI	*P* value
Age (1 year)	1.03	1.01–1.05	0.0016	1.01	0.99–1.03	0.22
Duration of RA (1 year)	1.02	1.00–1.04	0.078	1.00	0.98–1.02	0.89
DAS28-ESR	1.81	1.41–2.33	< 0.0001	1.70	1.30–2.22	< 0.0001
HAQ	7.69	4.3–13.8	< 0.0001			
MTX use	0.46	0.27–0.78	0.0039	0.47	0.26–0.84	0.0098
PSL use	2.07	1.23–3.48	0.0054	1.37	0.78–2.41	0.27
Biological agents use	0.90	0.57–1.42	0.66	1.02	0.62–1.68	0.94
Right hand grip	0.90	0.87–0.94	< 0.0001			
Left hand grip	0.91	0.88–0.94	< 0.0001			
Walking speed	0.04	0.013–0.103	< 0.0001			

Results of univariate logistic (left) and multivariate logistic (right) regression analysis with RA-related factors regarding the presence of prefrailty or frailty. A dummy variable was constructed as follows: 0 = robust and 1 = prefrailty or frailty, and these regression analyses were performed with its frailty variable as a dependent variable.

*RA* rheumatoid arthritis, *DAS28-ESR* 28-joint Disease Activity Score using erythrocyte sedimentation rate, *HAQ* health assessment questionnaire, *MTX* methotrexate, *PSL* prednisolone.

### Habitual fish intake is negatively associated with frailty status

**Figure 1:**
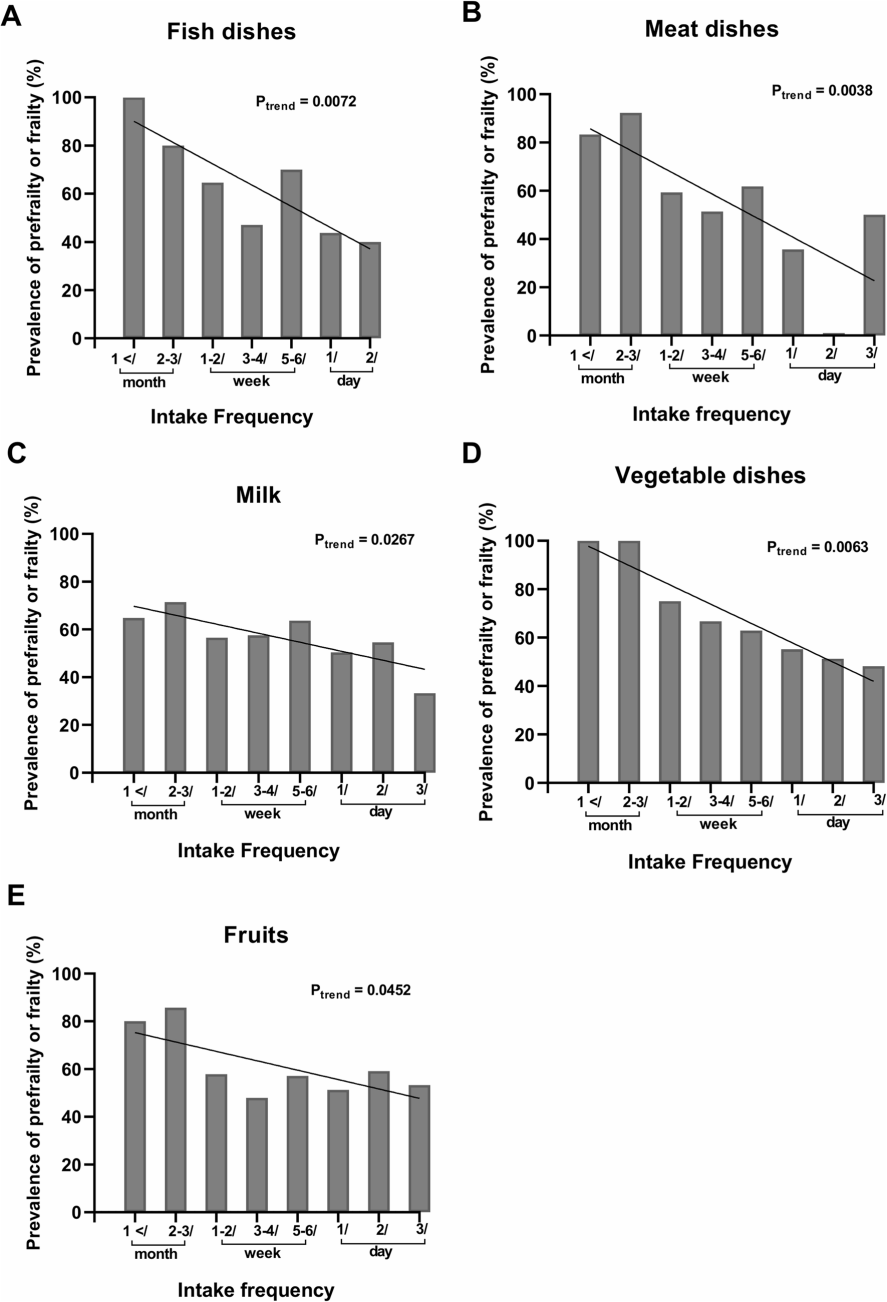
The prevalence of prefrailty or frailty for subjects by intake frequency. The vertical axis represents percentage of prefrailty or frailty in the categories of intake frequency. Five of the 20 groups are negatively correlated with the prevalence of prefrailty and frailty; fish (**A**), meat (**B**), milk (**C**), vegetable (**D**), and fruits (**E**). P_trend_ values were obtained from the results of Cochran-Armitage trend test.

Because little is known regarding nutrition therapy for frailty in RA in contrast to the general population, we performed analysis to determine the relationship between frailty with RA and food intake habits. Trend analysis revealed that the frequency of food intake was inversely correlated with the prevalence of prefrailty or frailty in 5 of the 20 groups on the questionnaire: fish (Fig. 1A), meat (Fig. 1B), milk (Fig. 1C), vegetable (Fig. 1D), and fruits (Fig. 1E). We then conducted multivariate logistic analyses with frail/prefrail as a dependent variable. In the model that includes RA-related factors and fish as covariates (Table 4 left), RA patients eating fish more than two times/week had a lower prevalence of frailty than those eating fish two times/week or less (OR 0.31: 95% CI 0.18–0.55). The same association was maintained in the model including RA-related factors and the source of protein (fish and meat) (Table 4 middle) as well as in the model including RA-related factors and 5 food groups (Table 4 right). DAS28-ESR, MTX use and age were also identified as independent covariates. These results were similarly observed when using a categorical variable dividing age by 75 years old, which has been widely reported to be associated with the prevalence of frailty (Supplementary Table 1). Furthermore, we obtained the same results even after using another logistic regression model, a forward stepwise logistic regression model (Supplementary Table 2). We next performed a similar analysis in elderly RA patients aged 65 years and older because clinical definitions of frailty generally targeted the elderly who were over 65 years of age, and might be inappropriate for such categorization of younger participants. As in the analysis of all RA patients, habitual fish intake more than two times/week was significantly associated with decreased prevalence of frailty (OR 0.20: 95% CI 0.062–0.62) even in the group of patients over 65 years old (Table 5). These results indicate that habitual fish intake is strongly associated with lower prevalence of frailty in RA patients.

**Table 4:** Multivariate logistic analysis for RA patients with prefrailty or frailty together with dietary habits.

	Model 1		Model 2		Model 3	
	OR (95% CI)	*P* value	OR (95% CI)	*P* value	OR (95% CI)	*P* value
DAS28-ESR	1.79 (1.35–2.37)	< 0.0001	1.78 (1.34–2.36)	< 0.0001	1.73 (1.30–2.30)	0.00020
MTX use	0.43 (0.23–0.79)	0.0052	0.43 (0.23–0.79)	0.0065	0.42 (0.23–0.78)	0.0060
Age (1 year)	1.03 (1.00–1.05)	0.023	1.02 (1.00–1.05)	0.038	1.03 (1.01–1.05)	0.0017
PSL use	1.22 (0.68–2.19)	0.50	1.23 (0.68–2.20)	0.49	1.21 (0.67–2.20)	0.52
Duration of RA (1 year)	1.00 (0.97–1.02)	0.65	1.00 (0.97–1.02)	0.66	1.00 (0.98–1.02)	0.79
Biological agents use	0.98 (0.59–1.64)	0.94	0.98 (0.58–1.63)	0.93	1.00 (0.59–1.67)	0.99
Fish dish	**0.31 (0.18–0.55)**	** < 0.0001**	**0.32 (0.18–0.57)**	** < 0.0001**	**0.35 (0.19–0.63)**	**0.00060**
Meat dish			0.86 (0.49–1.49)	0.58	0.89 (0.51–1.57)	0.69
Milk					0.72 (0.41–1.24)	0.24
Vegetable					0.94 (0.47–1.91)	0.87
Fruits					0.78 (0.42–1.43)	0.42

Results of multivariate logistic regression analysis with dietary habits and RA-related factors regarding the presence of prefrailty or frailty. Model was adjusted for age, RA duration, RA therapeutics (use of prednisolone, biologics and methotrexate), DAS28-ESR, and dietary habits (only fish dish (model 1), fish + meat dishes (model 2) and fish/meat/milk/vegetable/fruits (model 3) as variables).

Dummy variables were constructed for intake frequency as 0 and 1: Fish and Meat dish: 0 = low frequency (≤ 2 times/weeks), 1 = high frequency (3 times/week ≤) and Milk, Vegetable and Fruits: 0 = low frequency (≤ 6 times/week), 1 = high frequency (1 time/day ≤).

*RA* rheumatoid arthritis, *DAS28-ESR* 28-joint Disease Activity Score using erythrocyte sedimentation rate, *MTX* methotrexate, *PSL* prednisolone.

**Table 5:** Multivariate logistic analysis for RA patients over 65 years old with prefrailty or frailty including factors of dietary habits.

	Model 1		Model 2		Model 3	
	OR (95% CI)	*P* value	OR (95% CI)	*P* value	OR (95% CI)	*P* value
DAS28-ESR	2.17 (1.38–3.42)	0.00080	2.17 (1.38–3.42)	0.00080	2.15 (1.35–3.41)	0.0012
MTX use	0.28 (0.11–0.69)	0.0057	0.28 (0.11–0.69)	0.0057	0.26 (0.10–0.65)	0.0043
Age (1 year)	1.11 (1.02–1.21)	0.013	1.11 (1.02–1.21)	0.013	1.12 (1.03- 1.22)	0.011
PSL use	1.20 (0.51–2.80)	0.68	1.20 (0.51–2.80)	0.68	1.35 (0.55–3.31)	0.51
Duration of RA (1 year)	1.00 (0.97–1.03)	0.98	1.00 (0.97–1.03)	0.98	1.00 (0.97–1.03)	0.90
Biological agents use	1.06 (0.46–2.44)	0.90	1.06 (0.46–2.44)	0.90	1.09 (0.47–2.55)	0.85
Fish dish	**0.19 (0.066–0.57)**	**0.0029**	**0.19 (0.066–0.58)**	**0.0031**	**0.20 (0.062–0.62)**	**0.0054**
Meat dish			1.01 (0.45–2.27)	0.98	1.01 (0.44–2.36)	0.97
Milk					0.78 (0.30–2.05)	0.61
Vegetable					3.18 (0.88–11.5)	0.079
Fruits					0.48 (0.16–1.44)	0.19

Results of multivariate logistic regression analysis with dietary habits and RA-related factors regarding the presence of prefrailty or frailty in RA patients aged 65 years and older. Model was adjusted for age, RA duration, RA therapeutics (use of prednisolone, biologics and methotrexate), DAS28-ESR, and dietary habits (only fish dish (model 1), fish + meat dishes (model 2) and fish/meat/milk/vegetable/fruits (model 3) as variables). Dummy variables were constructed for intake frequency as 0 and 1: Fish and Meat dish: 0 = low frequency (≤ 2 times/weeks), 1 = high frequency (3 times/week ≤) and Milk, Vegetable and Fruits: 0 = low frequency (≤ 6 times/week), 1 = high frequency (1 time/day ≤).

*RA* rheumatoid arthritis, *DAS28-ESR* 28-joint Disease Activity Score using erythrocyte sedimentation rate, *MTX* methotrexate, *PSL* prednisolone.

## Discussion

To the best of our knowledge, the present study is the first to reveal statistical correlations between dietary habits and frailty in an RA population. The prevalence of frailty was higher compared to that in general^21^, and was significantly correlated with current disease activity (DAS28-ESR) in multivariate logistic analysis. These results accord with previous reports that RA patients have higher prevalence of sarcopenia than the community-dwelling elderly, and that pro-inflammatory cytokines such as IL-6 and TNF-α participate not only in RA progression but also in frailty development due to catabolic effects on muscles^9–11^. Inflammatory mediators are also associated with the development of several age-related diseases (i.e., dementia, Parkinson’s disease, atherosclerotic cardiovascular disease and type 2 diabetes)^22^, and may be candidates for therapeutic targets of frail progression.

In univariate logistic analysis, other variables were also associated with the presence of frailty such as age, physical activity parameters (hand grip strength, walking speed) and PSL/MTX use. Age-related decline in physical activity including hand grip strength and walking speed is the very definition of frailty, and these results suggest the validity of the SOF index for assessing frailty status in RA populations as well as in general. PSL use induces proximal muscle wasting and weakness, and is an indicator of thigh muscle area and density in RA patients. MTX use was negatively associated with the prevalence of frailty in the analysis, which might reflect a limitation of therapeutic options for frail patients^7^.

In additional multivariate analyses, we also found that fish intake frequency rather than meat intake frequency was negatively correlated with the presence of frailty in RA patients. Fish is well known as an important source of potentially beneficial nutrients for preventing frailty such as protein and n-3 polyunsaturated fatty acids (n-3 PUFAs). High protein intake is associated with low prevalence of frailty^13,23^, and fish oil–derived n–3 PUFA therapy slows the normal decline in muscle mass and function^24^. Serum levels of n-3 PUFA parallel dietary fish consumption^25,26^ and correlate with a 27% reduction in all-cause mortality risk of elderly^27^. Marine-derived n-3 PUFAs also have anti-inflammatory effects on RA progression^28^. In prospective, double-blind, randomized controlled studies by Kremer et al.^29,30^, dietary supplementation of n-3 PUFAs dose-dependently decreases RA activity parameters (tender joint count, swollen joint count and duration of morning stiffness) and serum levels of IL-1β. n-3 PUFAs also protect against joint inflammation in some animal models of RA^31–33^, and modulate a range of immunological reactions in RA patients such as reduced production of leukotriene B4 by neutrophils^29^, IL-1 by macrophage^29^ and prostaglandin E2 by mononuclear cells^34^. In addition, our group has previously shown a negative correlation between seafood intake frequency and RA disease activity^14^. Based on our results and these knowledge, habitual fish intake may contribute to the prevention of frailty through anti-inflammatory effects via n-3 fatty acid and partly through protein intake.

In the present study, we applied a combination of simplified frailty index (SOF criteria) and food frequency questionnaire (FFQ) to assess frailty and dietary habits. SOF criteria have only three components (weight loss, inability to do five chair stands, poor energy), and efficiently predict risk of adverse outcomes and mortality^35^. FFQ is a straightforward self-reported questionnaire previously used for quantitative evaluation of dietary habits in RA patients as well as in general^14,18^. Both are simple and versatile methods, and are potentially useful for investigation of correlations between frailty and dietary habits in various clinical populations.

There are several major limitations in the present study. Although the FFQ in the current study can determine the intake frequency of each type of foods, we cannot estimate the actual total amount of protein intake from the FFQ data and cannot determine whether the benefit of fish consumption is due to increased protein intake or other beneficial compounds. Our cross-sectional study does not imply causation, and the long-term relationship of dietary habits and frailty is still unknown. It is not known whether our findings can be generalized because of clinical factors that may differ in each study, such as epidemiological backgrounds of our participants, a dietary culture based on fish, and criteria of frailty. In addition, only female participants were included in the present study. Moreover, RA patients treated in a university hospital may have uncommon clinical characteristics compared to those in other medical institutions.

In conclusion, frailty has a positive correlation with RA disease activity and a negative correlation with fish intake frequency. Habitual fish intake may thus play a key role in nutritional intervention for preventing progression of frailty and RA.

## Supplementary Information

Supplementary Information
