# Supplementary material for: Habitual fish intake negatively correlates with prevalence of frailty among patients with rheumatoid arthritis
Source: Sci Rep. 2021 Mar 3;11:5104. doi: 10.1038/s41598-021-84479-0 (PMC7930016; doi:10.1038/s41598-021-84479-0)
Supplement: Supplementary file 1 — Supplementary Information [file 41598_2021_84479_MOESM1_ESM.docx]

**Habitual fish intake negatively correlates with prevalence of frailty among patients with rheumatoid arthritis**

Hiroto Minamino, Masao Katsushima, Mie Torii, Motomu Hashimoto, Yoshihito Fujita, Kaori Ikeda, Wataru Yamamoto, Ryu Watanabe, Kosaku Murakami, Koichi Murata, Kohei Nishitani, Masao Tanaka, Hiromu Ito, Koichiro Ohmura, Hidenori Arai, Nobuya Inagaki, Shuichi Matsuda

**Supplementary information:**

**Supplementary Table1. Multivariate logistic analysis for RA patients with prefrailty or frailty using a categorical variable with a cutoff point at 75 years old**

|  |  | |
| --- | --- | --- |
| **variables** | **OR (95% CI)** | ***P* value** |
| DAS28-ESR | 1.75 (1.32 - 2.33) | 0.00010 |
| Age (< 75,75≦) = (0,1) | 3.59 (1.43 - 9.01) | 0.0035 |
| MTX use | 0.44 (0.23 - 0.81) | 0.0089 |
| PSL use | 1.26 (0.70 - 2.29) | 0.45 |
| Duration of RA (1 year) | 1.00 (0.98 - 1.02) | 0.94 |
| Biological agents use | 1.04 (0.61 - 1.75) | 0.90 |
| Fish dish | **0.38 (0.22 - 0.69)** | **0.0013** |
| Meat dish | 0.83 (0.47 - 1.45) | 0.51 |
| Milk | 0.73 (0.42 - 1.27) | 0.26 |
| Vegetable | 0.95 (0.47 - 1.91) | 0.89 |
| Fruits | 0.86 (0.47 - 1.57) | 0.63 |

**Supplementary Table1.** Results of multivariate logistic regression analysis with dietary habits and RA-related factors regarding the presence of prefrailty or frailty using a categorical variable with a cutoff point at 75 years old. Dummy variables were constructed for age as 0 and 1 (0 = < 75 years old, 1 = 75 years old ≦).

Abbreviations: *RA* rheumatoid arthritis, *DAS28-ESR* 28-joint Disease Activity Score using erythrocyte sedimentation rate, *MTX* methotrexate, *PSL* prednisolone

**Supplementary Table2.** **Multivariate logistic analysis for RA patients with prefrailty or frailty using a forward stepwise logistic regression model**

|  |  |  |
| --- | --- | --- |
| **variables** | **OR (95% CI)** | ***P* value** |
| DAS28-ESR | 1.81 (1.38 - 2.38) | < 0.0001 |
| Age (1 year) | 1.03 (1.00 - 1.05) | 0.02 |
| MTX use | 0.42 (0.23 - 0.76) | 0.0045 |
| Fish dish | 0.31 (0.18 - 0.54) | < 0.0001 |

**Supplementary Table2.** Results of a forward stepwise logistic regression analysis with dietary habits and RA-related factors regarding the presence of prefrailty or frailty. We conducted a forward stepwise setting *P* for enter < 0.1 and exit > 0.1.

Abbreviations: *RA* rheumatoid arthritis, *DAS28-ESR* 28-joint Disease Activity Score using erythrocyte sedimentation rate, *MTX* methotrexate
